# Mathematics and malaria

**DOI:** 10.7554/eLife.00385

**Published:** 2012-12-18

**Authors:** Prabhat Jha

**Affiliations:** 1 **Prabhat Jha**, a senior editor on *eLife*, is in the Dalla Lana School of Public Health, and the Centre for Global Health Research, University of Toronto, Canadajhap@smh.ca

**Keywords:** parasite population structure, epidemiological dynamics, antigenic diversity, immune selection, Plasmodium falciparum, var multi-gene family, Other

## Abstract

Populations of malaria parasites are strongly influenced by their interactions with human immunity, and a better understanding of these interactions can help to explain why the risks from this potentially lethal disease vary according to location and age.

**Related research article** Artzy-Randrup Y, Rorick M, Day K, Chen D, Dobson A, Pascual M. 2012. Population structuring of multi-copy, antigen-encoding genes in *Plasmodium falciparum*. *eLife* **1**:e00093. doi: 10.7554/eLife.00093

**Image** Pathogens like *P. falciparum* display a high level of genetic diversity

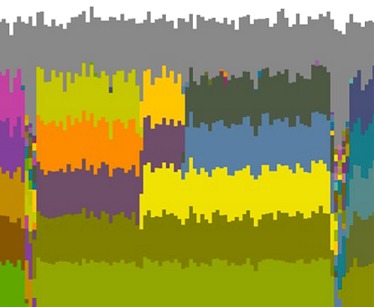

Parasites are a lot like teenagers. Take a few hundred adolescents in school, each with unique genetic and cultural influences, and you would expect to see huge diversity in behaviour. Instead, we generally find that teenagers fall into one of a small number of groups that have their own distinct identities and patterns of behaviour (or misbehaviour). Similarly, the genetic make-up of *Plasmodium falciparum*, the parasite that causes most fatal malaria cases, suggests that many different strains of malaria should exist in a host population: in reality, however, only a small number of strains, each with distinctive clinical and epidemiological features, are observed. Peer pressure is the driving force for a finite set of teenager behaviours (Brown et al., 1986), and the immune response of humans appears to be the driving force for the same effect in many pathogens.

Malaria, which arises from *P. falciparum* carried by mosquitos, is thought to kill more than a million people a year and to infect many more. *P. falciparum* causes malaria by entering red blood cells and releasing antigens that change the surfaces of the cells; these infected cells then accumulate in the brain and various small blood vessels. The virulence of *P. falciparum* is believed to arise from its ability to evade the human immune system by changing the antigens that are released. Many important pathogens, including *P. falciparum*, constantly exchange genetic material: however, the number of strains of malaria (and other diseases) is much lower than expected, given the level of genetic exchange that happens. This has led to ‘strain theory’—the idea that populations of parasites are restricted to specific subtypes (that is, different strains) as a result of their interactions with human immunity (Gupta et al., 1994, 1996). Now, writing in *eLife*, Yael Artzy-Randrup and co-workers at the University of Michigan, New York University and Princeton University extend strain theory and conclude that immune selection markedly influences populations of *P. falciparum*, restricting their diversity to a finite number of antigenic strains (Artzy-Randrup et al., 2012).

They focus on the family of 50–60 *var* genes that code for the PfEMP1 antigens that are central to malarial disease. Each parasite expresses a single *var* gene at a time, while the rest remain transcriptionally silent. However, as the hosts develop antibodies against the single antigen that is being produced, small sub-populations of *P. falciparum* begin to produce other types of antigens so as to re-establish the infection. Artzy-Randrup and co-workers developed an ‘agent-based model’ to describe interactions between parasites and humans through simple mathematical rules that quantify genetic and epidemiological processes (Bauer et al., 2009).

The team's model predicts that host immune selection can cause the local *P. falciparum* population to self-organise into a finite number of strains (see Figure 1), with the degree of genetic overlap between the strains (defined as the number of common *var* alleles) depending on the transmission intensity (defined as the number of bites per year). The strains do not, in general, have common *var* alleles, but partially overlapping *var* alleles can arise when the transmission intensity is high, as it is in many parts of Africa. High transmission intensities also lead to immunity being established in humans at earlier ages than happens in areas with lower transmission intensities, such as parts of Asia: the proportion of people who become immune is also higher in areas with high transmission intensities. The changes in the parasite population appear to be mostly a product of the traits of immune response of humans, while ‘neutral’ factors, such as genetic recombination from random mating, appear to have much less influence.

**Figure 1.**
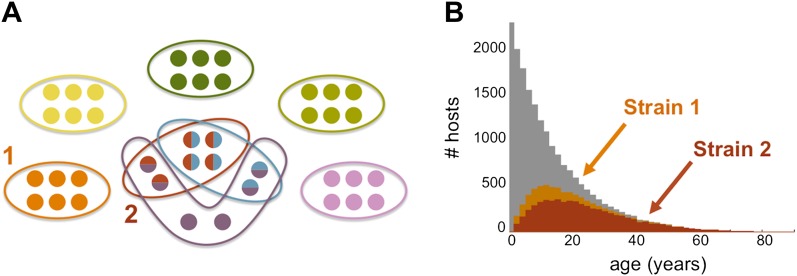
The genome of *P. falciparum* contains between 50 and 60 *var* genes that code for different antigens, all of which can lead to malaria. Despite this diversity, only a relatively small number of strains of malaria become established in any population: moreover, these strains tend to have little or no genetic overlap with each other. (**A**) This illustration (created by Artzy-Randrup et al. and motivated by real data) shows how 40 *var* genes (circles) can lead to 8 different strains of *P. falciparum* malaria (each composed of a subset of 6 genes). Five of these strains are unique as they do not genetically overlap with any of the other strains; two strains (shown in red and light blue) share all of their genes with other strains, and one strain (shown in purple) shares four of its genes with other strains. (**B**) The number of hosts immune to two strains of malaria versus age: strain 1 (orange) is genetically unique, so immunity to this strain is gained only through exposure to it; strain 2 (red) shares genes with other strains of malaria, so immunity to this strain can be acquired through exposure to it and also through exposure to two other strains of malaria. Immunity to strain 1 is higher than for strain 2 as human hosts are exposed to strain 1 at a higher level and at younger ages than strain 2. The grey bars show the host age distribution.

FIGURE: ARTZY-RANDRUP ET AL.

It has long been recognized that malaria infection in humans varies greatly from place to place, and it has been assumed that these differences arise from biological differences in otherwise morphologically similar parasites. As early as 1910, Ronald Ross—the British doctor who won the Nobel Prize in Physiology or Medicine in 1902 for demonstrating that mosquitos transmit the parasites that cause malaria—had noted different types of fever in malaria patients. Then, in work that was just as influential as the work that won him the Nobel Prize, Ross developed mathematical models to describe the spread of malaria (Ross, 1916), which eventually paved the way for the spraying-based strategies that have been widely used in an effort to stop the spread of the disease (see McKenzie et al., 2008 for an historical review).

The biological differences in *P. falciparum* presumably lead to large variations in its ability to infect mosquitos (only some species of mosquitoes can acquire the parasite), in the patterns of human infection, and in its susceptibility to drugs (Snow et al., 2012). The suggestion by Artzy-Randrup et al. that lower intensity transmission is linked with loss of immunity might be consistent with recent findings from India which suggest that malaria kills substantial numbers of rural adults who lack access to curative therapies (Dhingra et al., 2010).

Mosquito nets treated with insecticides, spraying insecticides inside houses and, most importantly, drugs (notably artemisinin-combination therapies) have proved to be powerful tools in the fight against malaria (Breman et al., 2006). Strain theory suggests that vaccines should be possible, but an effective malaria vaccine has yet to be found: indeed, a recent trial of the RTS,S/AS01 vaccine found that it provided only ‘modest protection against both clinical and severe malaria in young infants’ (The RTS,S clinical trials partnership, 2012).

Malaria is increasingly the subject of attention around the world, and a better understanding of the basic biologic processes involved will be central to developing the tools which can better control this lethal, but curable disease. More than a century after the pioneering work of Ross, malaria still presents a formidable challenge to medical science.
